# Numerical Study of Joule Heating Effects on Microfluidics Device Reliability in Electrode Based Devices

**DOI:** 10.3390/ma14195819

**Published:** 2021-10-05

**Authors:** Caffiyar Mohammed Yousuff, Vineet Tirth, Mohamed Zackria Ansar Babu Irshad, Kashif Irshad, Ali Algahtani, Saiful Islam

**Affiliations:** 1 Department of Electronics and Communication Engineering, C. Abdul Hakeem College of Engineering and Technology, Melvisharam 632509, India; zackriaansarbi@gmail.com; 2 Mechanical Engineering Department, College of Engineering, King Khalid University, Abha 61411, Saudi Arabia; vtirth@kku.edu.sa (V.T.); alialgahtani@kku.edu.sa (A.A.); 3 Research Center for Advanced Materials Science (RCAMS), King Khalid University, P.O. Box 9004, Abha 61413, Saudi Arabia; 4 Interdisciplinary Research Center for Renewable Energy and Power Systems (IRC-REPS), King Fahd, University of Petroleum & Minerals, Dhahran 31261, Saudi Arabia; 5 Civil Engineering Department, College of Engineering, King Khalid University, Abha 61411, Saudi Arabia; sfakrul@kku.edu.sa

**Keywords:** microfluidics, DEP based devices, joule’s heating, device reliability, cell separation

## Abstract

In electrode-based microfluidic devices, micro channels having narrow cross sections generate undesirable temperature inside the microfluidic device causing strong thermal distribution (joule heating) that eventually leads to device damage or cell loss. In this work, we investigate the effects of joule heating due to different electrode configuration and found that, electrodes with triangular arrangements produce less heating effect even at applied potential of 30 V, without compromising the performance of the device and separation efficiency. However, certain electrode materials have low thermal gradients but erode the channel quickly thereby affecting the reliability of the device. Our simulation also predicts optimal medium conductivity (10 mS/m with 10 V) for cells to survive inside the channel until they are selectively isolated into the collection outlet. Our investigations will aid the researchers in the designing of efficient and reliable microfluidic devices to overcome joule heating inside the microchannels.

## 1. Introduction

The synergies between electrokinetics and dielectrophoresis offer unique capabilities to drive the particles or cells in microchannel by inducing applied electric field externally. Dielectrophoresis (DEP) is a technique to manipulate cells in precise ways which enables label-free sorting of cells from heterogenous mixtures of population and enables scientific inquiry that cannot be addressed using conventional methods [1,2]. However, joule heating is often observed in microchannel in electrode-based microfluidics device such as DEP. When electric field is applied to induce particle motion in a microchannel, the electric current passing through the medium having conductivity results in joule heating [3]. Numerous attempts have been made to understand, how generation of joule heating can be positively utilized in electrode-based devices for cell manipulation and analysis [4,5,6,7,8,9,10,11,12,13,14,15]. However, the driven electric field can cause catastrophic side effects inside a microchannel due to the heat dissipated in the medium through which electric current is passed. Since microfluidic device has very narrow channel geometry and due to the fact that electrical resistance is more as we decrease the channel cross section, more heat is produced inside a channel with increased resistance. This results in temperature gradient formation in the fluid present inside the microchannel. This elevated temperature may denature the biological entities present in the samples and also makes the liquid properties non-uniform such as its electrical conductivity, viscosity, and thermal conductivity of the liquid [16]. The variations and disturbances resulting from joule heating may inevitably affect throughput and reliability of the DEP-based devices [17]. 

Therefore, it is important to study the joule heating to understand its effects in device performance and cell health [18], especially, in applications to cell sorting and manipulation, by varying various parameter constraints in order to come out with the optimal design and efficient operation of microfluidic systems. Several DEP-based microfluidic devices were developed by focusing on various design constraints, such as electrode geometries, channel geometries to investigate the efficacy of sorting [19,20,21,22,23]. The potential impact of joule heating on device reliability and performance have been widely studied and demonstrated by several researchers [24,25,26,27,28] in applications to cell manipulations and isolations. However, the studies do not demonstrate the effects of different parameters that actually contribute to joule heating effect. Therefore, in our study, we found that, the electrode arrangements and the choice of electrode geometries generally requires large applied potentials to produce sufficient DEP force for effective separation of cells/particles [4,29,30]. These large potentials create strong electric field gradients for particle separation, but at the same time, it creates strong joule heating region.

An electrode with tip edges (triangular electrode) requires less applied voltage as compared to flat rectangular/square electrode edges, that can generate a strong DEP force required for effective cell separation [31,32]. However, it creates a strong thermal gradient across the micro channel, especially around the electrode tip thereby creating strong joule-heating region. The exposed cells in these regions eventually get exposed to this high temperature which causes damage to the cell if the threshold temperature exceeds over 316 K [33]. We observed that, there are various factors that lead to joule heating problem in microfluidic devices such as electrode geometry, electrode potentials, substrate materials, and conductivity of the medium. Therefore, in this study, we attempt to explore the effects of these parameters which can provide optimal solution to avoid joule heating effects thereby improving device performance and reliability with no or less cell damage. In our findings, we observed the effects of temperature on the cell when it is exposed to the region of strong thermal gradient at various positions in a microchannel (initial, center, and rear end). The thermal impact on cells and its survival time provides a comprehensive understanding about cell behavior. We explore the optimum design constraints to ensure effective separation and cell viability in DEP-based microfluidic devices. This study will enable the future researchers to develop a reliable and effective DEP device and other electrode-based microfluidic devices for various bio clinical applications.

## 2. Materials and Methods

### 2.1. Theory of DEP

**Figure 1:**
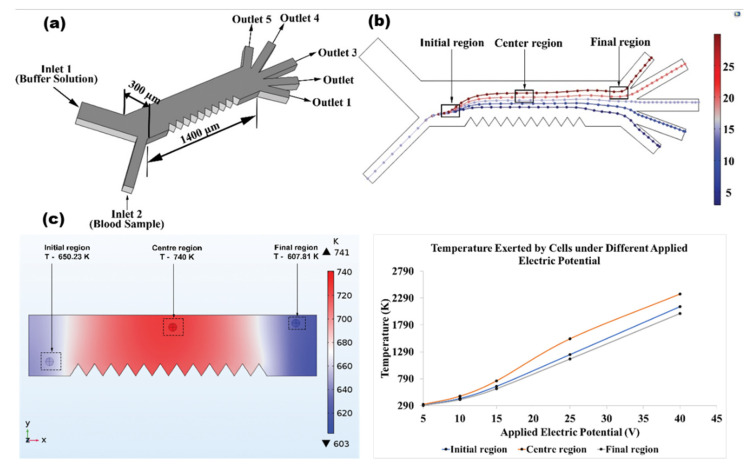
A microfluidic chip design with five outlets arrangements was successfully simulated to separate multiple targets in a single run. (**a**) The schematic illustration of five outlets device showing the dimensions with triangular electrodes arrangements in the side wall. (**b**) Simulation of particle streamlines reveals that cells of distinct sizes (25, 20, 15, 10, 7 µm) were selectively collected in target reservoirs. (**c**) Simulations verify that, strong joule heating was observed in center region of the channel where electric field gradient is very strong.

DEP force is generated due to particle exposure to non-uniform electric field. The amount of force experienced by the particle is dependent on various parameters like applied potentials, conductivity of the medium, and size of the particles [34] (see equation 1). The device geometry is illustrated in Figure 1a, which shows that the cells of five different types can be selectively deflected to distinct collection outlets using DEP force generated through a series of triangular electrode arrangements at one side of the channel(+V) and flat electrode at the other side of the channel (-V). The simulation of separating five different cell types (25, 20, 15, 10, 7 µm) into different collection outlets is shown in Figure 1b.

F_DEP(i)_ = 2πε_0_ε_m_Re[*f_CM_* (ω)] R^3^ ∇E^2^ (1)

where “ε_m_” is the relative permittivity of the suspending medium, R is the radius of the particle, ∇E^2^ denotes gradient of the electric field, Re[*f_CM_* (ω)] denotes the real part of the Claussius Mosotti [CM] factor that determines effective polarizability and is expressed as[35],

(2) $$f_{CM}\left({\tilde{\varepsilon }}_{p},{\tilde{\varepsilon }}_{m}\right)=\frac{{\tilde{\varepsilon }}_{p}-{\tilde{\varepsilon }}_{m}}{{\tilde{\varepsilon }}_{p}+2{\tilde{\varepsilon }}_{m}}$$

where $\tilde{\varepsilon }$ is the complex permittivity and defined as,

(3) $$\tilde{\varepsilon }=\varepsilon -j\left(\;\frac{\sigma }{\omega }\;\right)$$

Therefore, *CM* factor can be written as,

(4) $$f_{CM}\left(\varepsilon_{p},\;\sigma_{p},\varepsilon_{m},\sigma_{m},\omega \right)=\frac{\left(\varepsilon_{p}-\varepsilon_{m}\right)+j/\omega \left(\sigma_{p}-\sigma_{m}\right)}{\left(\varepsilon_{p}+2\varepsilon_{m}\right)+j/\omega \left(\sigma_{p}+2\sigma_{m}\right)}\;\;$$

The *CM* factor is dependent on the complex permittivities of the particles and the suspending medium. 

### 2.2. Numerical Modeling for Joule Heating

With reference to equation 5, joule heating in the device is majorly due to applied potentials, field distribution, and conductivity. Therefore, change in any of these parameters has great influence on the thermal distribution across the microchannel of the device. In our study, we observed the effects of strong temperature distributions at the center region (200 µm from electrode wall, near electrode region), while at initial and final region, the effects are only minimal (Figure 1c). The effect of temperature at initial, final, and center regions was simulated for varying potentials and we found that, temperature distribution at the center region is very strong and hence the joule heating is more at this region. Therefore, we have considered the center region as the main region of interest throughout our study.

If “*σ*” is the conductivity of the buffer solution between the positive and negative electrode then the heating power per volume *V* depends linearly to conductivity of the suspending medium (*σ*) and to the square of the electric field strength and is expressed as [36],

(5) $$q=\frac{P_{heat}}{V}=\sigma \;.\;E_{rms}^{2}\;\propto \;\sigma \;.\;U_{rms}^{2}\;$$

where “*rms*” denotes root mean square value, “*U*” represents the voltage applied between the electrodes. The amount of heat generated (*P_heat_*) is equal to the amount of heat dissipated under the steady state condition [3]. The electric field (*E_rms_*) depends on the conductivity of the suspending medium (*σ*) which is greatly affected by the change in temperature and is expressed as,

σ = σ_0_[1 + *α*(*T* − *T*_0_)] (6)

Here, “σ_0_” refers to the conductivity at a reference temperature *T*_0_, and α is the temperature coefficient of the suspending medium. The Joule heating-induced temperature field is governed by the energy equation expressed as [37],

(7) $$\rho {∁}_{p}\left(\frac{\partial T}{\partial t}+\vec{u_{B}}\;\nabla T\right)=k_{1}\nabla^{2}T+\lambda \left(T\right){\left(\nabla \phi \right)}^{2}$$

where ∁_p_ and *k*_l_ represents the specific heat and thermal conductivity of the buffer solution, respectively, and they are considered to be constant (i.e., independent of temperature).

### 2.3. Device Design and Simulation Studies

The blood cells mixtures are flown from of the inlets (inlet 2), while the buffer solution is flown through inlet 1 which will squeeze the cells from inlet 2 close to the electrode region [38]. The device has a channel length of 1400 µm and channel width of 300 µm. The electrolyte (PBS solution) in the ratio of 1:9 with respect to blood samples, was used throughout the simulation. The device has a thickness of 80 µm as seen in Figure 1a. Electric potential is applied on either side of electrode arrangements made at the channel wall for generating non-uniform electric field. +V is applied on the one side of the channel wall (triangular electrode) and –V is applied on the other side of the channel wall (flat electrode). Under the application of electric field, cells of different sizes experiences different dep forces [18] enabling them to selectively deflect cells to distinct collection reservoir.

COMSOL Multiphysics simulation software was used to simulate our study for realizing temperature distribution and joule heating effects for device reliability. Joule heating modelling requires coupling of following three physics interfaces (Fluid Flow, Electric Current, and Bio-Heat transfer in fluids):

(i) Electric current interface is used to generate non-uniform electric field within the microchannel.
(ii) (Bio-heat transfer interface in simulation is used to generate the thermal distribution on the blood cells with boundaries set to room temperature of 290 K. Laminar flow interface is used to enable fluid flow at desired velocity from both inlets (inlet 1 and inlet 2).
(iii) A time-dependent solver is used to solve electric field and thermal distribution that provides understanding of the results through joule heating simultaneously.

## 3. Results and Discussions

The main contribution of this paper is to identify optimal combinations of electrode geometry, applied potentials, electrode materials, and buffer conductivity that will lead to effective separation of particles/cells while observing less joule heating effects, in the case of DEP-based cell sorting device. Though our simulation results will only predict the optimal solution for DEP based device, the efficiency of our proposed solution may be further explored by experimental demonstration and other electrode-based device falling under this regime.

### 3.1. Contribution of Electrode Shapes in Producing Optimum Joule Heating

**Figure 2:**
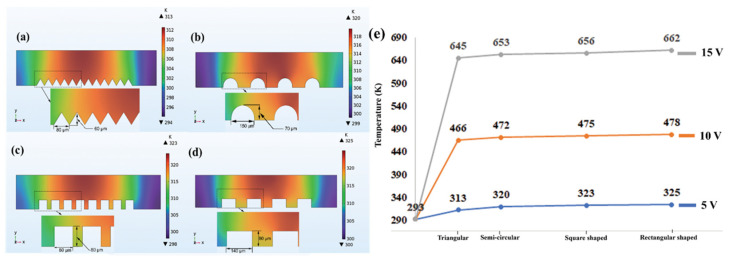
Simulation studies representing contribution of joule heating effects on various electrode shapes. (**a**) Triangular-shaped electrode generates fewer thermal gradients while maintaining reliable separation. The legend graph shows, temperature is around 310 K at the applied potential of 10 V. The insets are magnified views of electrode geometries showing the dimensions of the electrodes. (**b**) With same potential (10 V), semi-circular shape electrodes generate strong electric field leading to high temperature distribution inside the channel (around 322 K). (**c**) The square shaped electrode generates electric field more than semi-circular geometry (above 327 K). (**d**) Rectangular shaped electrode geometry produces even more electric field than square electrode (330 K) at 10 V. (**e**) The comparison plots reveal that triangular electrodes remain attractive even if applied potential is varied between 5 and 15 V, to keep the survival time of cells within threshold as compared to other electrode geometry, however increasing voltage beyond 15 V produces high joule heating (temperature beyond 400 K damages cell).

We performed simulation to investigate the effects of joule heating on various electrode shapes (triangular, square, rectangle, and semi-circular geometry) to come out with efficient design for reliable DEP separation device. The reliability of the device performance was found to be much better when triangular electrode arrangements are employed. From our study, we found that, complete cell separation can be easily achieved with time period of 1 s (time for samples to flow from inlet to outlet). Using triangular shaped electrode with applied potential of 5 V, we observed much stronger DEP forces compared to square and rectangular while generating low thermal gradient at the center region (the temperature measured was relatively low as close to 310 K (Figure 2a,e), where the cells managed to survive for at least 1.45 s inside the device, which is sufficient enough for effective separation). However, under the same voltage, the temperature for semicircular, square, and rectangular electrodes was observed to be 322 K, 327 K, and 330 K (Figure 2b–d) which may eventually cause cell damage as it is greater than the threshold temperature (>316 K) for cell survival. We further noticed that the device with triangular geometry remains reliable for separation, even with applied potentials varying between 10 V and 25 V (Figure 2e). However, with other electrode geometries, beyond 10 V, the temperature drastically increases over 600 K (which is above the threshold value of the cell withstanding temperature) and cell survival time is only 0.3 s, 0.1 s, and 0.04 s respectively (much less than the required time for flow from inlet to outlet), which may denature the cell condition.

### 3.2. Choice of Electrode Materials Affects Reliablity of the Device

**Figure 3:**
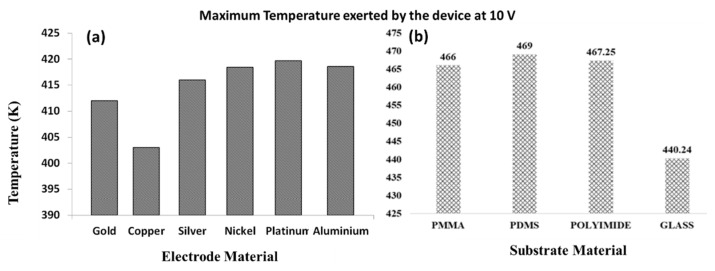
Simulation predicts that, choice of different electrode materials and substrate materials causes strong thermal distributions eventually affecting reliability and performance of the sorting system. (**a**) Simulation graph reveals that copper electrode has least thermal distributions (less joule heating) even at the applied potential of 10 V, however increasing the potential erodes the channel quickly compared to gold and platinum. (**b**) At the applied potential of 10 V, only glass has very least thermal distribution and joule heating effects for effective performance of the device.

Not only the shapes, the choice of electrode materials also plays a key role in joule heating generation inside a microchannel. Therefore, our study was extended to investigate the effect of different electrode material which contributes to device performance and reliability. Materials such as gold, copper, silver, nickel, platinum, and aluminum are mostly used as electrode in microfluidic devices [39]. Since effective separation at low voltage of 5 V was demonstrated in previous section (Section 3.2), the simulation was performed on different electrode materials at this voltage range and change in temperature on each electrode was observed. The performance of the electrode for multiple runs was evaluated at 3 s. When the voltage was increased to 10 V, the device temperature increased to above 400 K. We observed ~10 K temperature variation in all the other electrodes compared to copper (see Figure 3a). Though copper electrode showed the least temperature distribution across the channel but due to electrode–fluid interaction, there is additional increase in device temperature when copper materials is used [14] and this will degrade and destroy the channel compared to gold and platinum. However, at 50 V, the temperature increases above 2000 K, which is quite high and may eventually lead to device failure. Further, we also observed that, change in different substrate materials causes variation in temperature distribution inside the microfluidic device that will ultimately contribute to joule heating effects. From Figure 3b, we noticed that glass substrate has the potential to generate the least amount of joule heating of 310 K compared to other substrate materials (PDMS, PMMA, polyimide).

### 3.3. Change in Buffer Conductivity Affects Survival Time of the Cells

**Figure 4:**
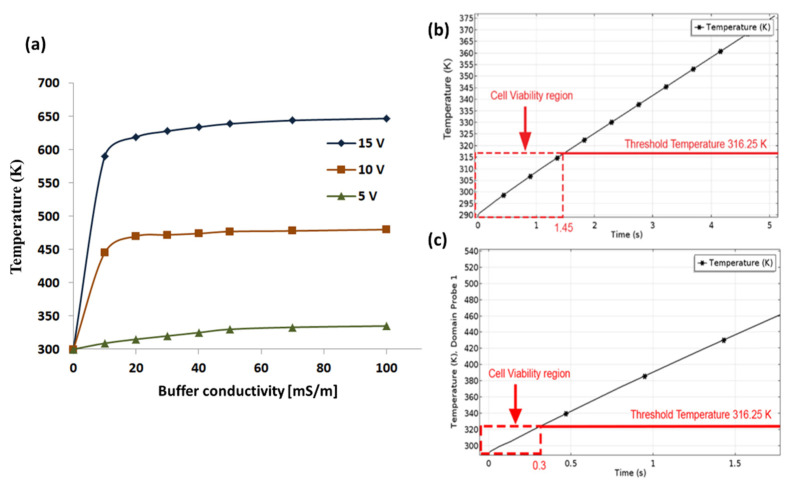
Simulation studies showing the effect of temperature at different conductivity level of the medium [10 mS/m to 100 mS/m]. (**a**) The plot of the temperature variation inside the channel reveals that, increases in conductivity causes increase in temperature at an applied potential of 5 V to 15 V. (**b**) While keeping buffer conductivity at 10 [mS/m] and applied potential of 10 V, the survival time of cells inside micro channel is found to be 1.45 s. This duration is sufficient enough to selectively deflect the cells to target outlets. (**c**) When the buffer conductivity is increased from 15 mS/m to 55 mS/m, the survival time is reduced to 0.3 s–0.1 s, this duration is very less for cells to survive inside the channel and will cause cell loss.

Our observation also provides a solution to optimize buffer conductivity of the medium for effective and reliable separation. The buffer conductivity varied from 10 [mS/m] to 100 [mS/m] to understand the effects of joule heating and reliable cell separation. We found that, thermal distribution inside the channel increases as conductivity and potential increase (see Figure 4a). However, with buffer conductivity between 10 [mS/m] and 30 [mS/m] and applied potential of 5 V, the temperature generated inside the channel is only 300 K (below threshold) and the survival time of cells inside the microchannel is found to be 1.45 s (Figure 4b). These values are sufficient for the cells to selectively deflect to target outlets without any loss or damage. When buffer conductivity is increased to higher values (40 [mS/m] to 100 [mS/m]), the temperature experienced by the cells increases over 325 K to 400 K (above the threshold value), causing cellular damage. To sort multiple cell types which require using high voltages (10 V, 15 V, 25 V, and 40 V), we found that, survival time of cells are found to be decreasing (0.3 s, 0.15 s, 0.04 s, and 0.1 s respectively) (see Figure 4c), which is quite less time for the cells to flow out of the device timely and eventually causing cellular damage.

## 4. Conclusions

The aim of this study is to explore the optimal parameters to design electrode-based dielectrophoretic device in order to minimize joule heating effects. It was observed that, the strong temperature distribution created inside a microchannel due to the variations of different parameters affects the performance and reliability of the device. We further noticed that temperature distribution is maximum at the center region and cells are likely to get damage at this region as it exposes to strong thermal gradients. Therefore, this region was considered as the region of interest throughout our study. 

From this work, the following main conclusions can be drawn:

- The triangular electrode is found to be effective in generating low thermal gradient at the center region, while maintaining microfluidic device reliable for separation, even at applied potential varied between 10 V and 40 V.
- Among different electrode materials considered, we found that copper generates low thermal gradient compared to other materials. However, copper electrodes degrades and destroys the channel as opposed to gold and platinum, and hinders its application for multiple runs.
- The device material fabricated using glass substrate has potential to generate least amount of joule heating, 313 K, compared to other substrate materials (PDMS, PMMA, polyimide) thereby increasing throughput of the separation system.
- With buffer conductivity of 10 [mS/m] and applied potential of 5 V, the survival time of cells inside microchannel is found to be 1.45 s. This duration is sufficient enough to selectively deflect the cells to target outlets. However, survival time was found to be decreasing with increasing buffer conductivity (upto 55 [mS/m]) making the device less reliable.

Hence, we anticipate that, our proposed guidelines will enable the researcher to design efficient and reliable microfluidic devices where joule heating is the main concern inside a microchannel.

## Data Availability

Data sharing is not applicable for this article.
